# Collaboration challenges in systematic reviews: a survey of health sciences librarians

**DOI:** 10.5195/jmla.2017.176

**Published:** 2017-10-01

**Authors:** Joey Nicholson, Aileen McCrillis, Jeff D. Williams

## Abstract

**Objective:**

While many librarians have been asked to participate in systematic reviews with researchers, often these researchers are not familiar with the systematic review process or the appropriate role for librarians. The purpose of this study was to identify the challenges and barriers that librarians face when collaborating on systematic reviews. To take a wider view of the whole process of collaborating on systematic reviews, the authors deliberately focused on interpersonal and methodological issues other than searching itself.

**Methods:**

To characterize the biggest challenges that librarians face while collaborating on systematic review projects, we used a web-based survey. The thirteen-item survey included seventeen challenges grouped into two categories: methodological and interpersonal. Participants were required to indicate the frequency and difficulty of the challenges listed. Open-ended questions allowed survey participants to describe challenges not listed in the survey and to describe strategies used to overcome challenges.

**Results:**

Of the 17 challenges listed in the survey, 8 were reported as common by over 40% of respondents. These included methodological issues around having too broad or narrow research questions, lacking eligibility criteria, having unclear research questions, and not following established methods. The remaining challenges were interpersonal, including issues around student-led projects and the size of the research team. Of the top 8 most frequent challenges, 5 were also ranked as most difficult to handle. Open-ended responses underscored many of the challenges included in the survey and revealed several additional challenges.

**Conclusions:**

These results suggest that the most frequent and challenging issues relate to development of the research question and general communication with team members. Clear protocols for collaboration on systematic reviews, as well as a culture of mentorship, can help librarians prevent and address these challenges.

## INTRODUCTION

Systematic reviews are an increasingly common research method used to compile and analyze large sets of existing study data from different sources. While thoughts about evidence synthesis date back to the late 1800s [1], the current focus on systematic reviews began mainly with Archie Cochrane, who used the phrase in the foreword to a 1989 book compiling evidence for pregnancy and childbirth interventions [2]. Shortly thereafter in 1992, the Cochrane Centre was formed, and one of its aims was to make it easier to conduct systematic reviews of trials [3]. Initially, systematic reviews were used to answer questions of effectiveness related to treatment or diagnosis of conditions. However, this methodology has now been expanded and used for an ever-increasing range of topics both within and outside of health care [1, 4], such as feasibility or appropriateness of care [5], crime and justice [6], and software engineering [7].

Since 1989, there has been ever-increasing use of systematic reviews as a research method. One estimate correctly predicted that at least 4,000 reviews would be published annually by 2010 and that this number would continue to increase [8]. With the increase in systematic reviews, there has also been an increase in recommended approaches to conducting this kind of research. The Cochrane Collaboration, the National Academy of Medicine (formerly, Institute of Medicine), and the Centre for Reviews and Dissemination, among others, have put out guidelines for researchers to follow when embarking on a systematic review. All of these guidelines either require or recommend collaborating with an experienced librarian to create a proper search strategy and help manage the methods [4, 9, 10].

There is a robust body of literature examining librarian involvement in the systematic review process, including articles focusing on emerging roles of librarians [11–13] and how librarian involvement improves review quality [14–17], improves search accuracy [14, 16, 18, 19], and increases retrieval of grey literature [16, 18, 20]. However, there has been little research on the challenges that librarians face in completing these projects and how to solve the challenges, specifically around methods other than the search itself and interpersonal issues.

While many librarians have been asked to participate in systematic reviews with local researchers, often these researchers are not familiar with the systematic review process or the appropriate role for librarians. The purpose of this study is to identify the challenges and barriers that librarians face when collaborating on systematic reviews. Because there is already a body of literature on librarian involvement in the search part of the process [14–20], the authors have deliberately focused on interpersonal and methods issues that span the rest of the systematic review process. The research questions are: (1) what challenges and barriers do health sciences librarians face when collaborating with researchers on systematic reviews?; (2) what are the most common and difficult challenges health sciences librarians faced when collaborating on systematic reviews?; and (3) what strategies do health sciences librarians employ to overcome these challenges?

## METHODS

### Instrument and study design

To measure and characterize the biggest challenges that librarians face while collaborating on systematic review projects, we used a web-based survey designed in Qualtrics survey software (Qualtrics, Provo, UT). The thirteen-item survey consisted of screening questions and questions related to demographics, training, and challenges associated with systematic reviews, as well as strategies used to overcome those challenges. An online survey was selected as the best method for describing the characteristics of this population since it allowed us to gather more results from a wider range of participants.

We identified seventeen challenges based on our own experiences in working on systematic reviews and then grouped these challenges into two categories: methodological and interpersonal. Participants were required to indicate the frequency with which they encountered the challenges described in the survey using a Likert scale composed of the values “Often,” “Sometimes,” “Rarely,” “Never,” and “Not sure.” Participants then ranked the top five challenges that they found most difficult to handle out of the full list of challenges to determine whether there was a relationship between how frequently the identified challenges were encountered and their perceived difficulty. Open-ended questions allowed survey participants to describe challenges not listed in the survey. Based on the diversity of experience and variety of strategies used to overcome challenges at our own institution, we chose to only have open-ended questions for participants to report their strategies.

After we developed a pilot survey based on our experiences, the survey was pretested for face validity by six health sciences librarians who were experienced in systematic reviews from New York University, the University of Iowa, the University of Maryland–Baltimore, and Weill Cornell Medical College. The survey was finalized based on their feedback (supplemental Appendix A).

### Population selection and recruitment

Participants were eligible to complete the survey only if they identified as a librarian who worked with a health sciences clientele and had worked on at least one systematic review. An email invitation with a link to the web-based survey was sent to prospective participants on April 7, 2015, via three professional email lists: MEDLIB-L, ExpertSearching, and AAHSL-all. A reminder email was sent to the same lists on April 21, 2015, one week before the survey closed. As an incentive to participate, survey respondents could provide their emails in a separate form to be entered in a chance to win one of two $25.00 Amazon.com gift cards. No identifying information was collected, and participants who entered the gift card drawing could not be linked to their specific survey responses. The researchers received exemption status for the study from New York University (NYU) School of Medicine Institutional Review Board prior to survey distribution.

### Analysis

Descriptive statistics were summarized using SPSS, version 23 (Chicago, IL). Where respondents were asked how frequently they had experienced a variety of challenges related to collaborating on systematic reviews (“Never,” “Rarely,” “Sometimes,” “Often,” “Not sure”), we collapsed responses into three variables for analysis. The options “Often” or “Sometimes” were combined to represent a common challenge. The options “Rarely” or “Never” were combined to represent a rare or nonexistent challenge. The remaining option “Not sure” could represent that the respondent was unsure of the meaning or how to respond. Open-ended responses were imported into Dedoose (Los Angeles, CA), and a thematic analysis was performed using a grounded theory approach to identify common themes.

## RESULTS

A total of 288 respondents began the survey. Thirty-five respondents did not meet the eligibility criteria and were excluded via the screening questions, and 54 respondents only partially completed the survey. The analyses were limited to the 199 respondents who met the inclusion criteria and fully completed the survey. The survey was distributed widely via professional email lists, so it was not possible to calculate a response rate.

Demographic details of the study population are described in Table 1. Over 80% of respondents were female of varying age. Years of professional experience as a health sciences librarian varied, but over half of respondents reported 11 or more years. Similarly, the number of systematic review projects completed also varied, with nearly half reporting having completed 6 or more. Only a small portion reported working on searches alone as opposed to being involved in more steps in the systematic review process. The majority of respondents reported having had training related to systematic reviews.

**Table 1:** Demographic details of the study population (n=199)

**Characteristic**	**n**	**%**
Gender		
Male	30	15.1%
Female	160	80.4%
Other/Prefer not to say	9	4.5%
Age range (years)		
20–29	12	6.0%
30–39	44	22.1%
40–49	52	26.1%
50–59	55	27.7%
60 or older	32	16.%1
Prefer not to say	4	2.0%
Years of experience as health sciences librarian		
1–5	46	23.1%
6–10	44	22.1%
11–15	40	20.1%
16–20	25	12.6%
21–25+	44	22.1%
Any systematic review training		
Yes	159	79.9%
No	40	20.1%
Number of systematic reviews completed		
Only assisted with search	32	16.1%
1–2	45	22.6%
3–5	24	12.1%
6–8	23	11.6%
8+	75	37.7%

A ranked list of all challenges listed in the survey is provided in Table 2. Of the 17 challenges, 8 were reported as common by over 40% of respondents. Systematic review methodology challenges were reported the most frequently across these areas: question is defined too broadly, researcher has no defined eligibility criteria, research question is not clear and answerable, researcher does not follow systematic review methods, researcher is not using two screeners, and question is defined too narrowly. This focus on methodology issues as a common challenge was echoed in the free responses, where 44 comments were received that discussed understanding or applying methods.

**Table 2:** Most frequently reported challenges in conducting systematic reviews reported by librarians (n=199)

**Type**	**Challenge**	**Often; Sometimes**		**Rarely; Never**		**Not sure**	
		
		**(n)**	**%**	**(n)**	**%**	**(n)**	**%**
Methods	Research question is defined too broadly (i.e., search retrieves more results than researcher wants to screen)	174	87.4%	22	11.1%	3	1.5%
Methods	Researcher does not have inclusion/exclusion criteria established at the beginning of process	153	76.9%	38	19.1%	8	4.0%
Methods	Research question is not clear and answerable	152	76.4%	41	20.6%	6	3.0%
Methods	Researcher does not follow systematic review methodology (e.g., doing a narrative review)	136	68.3%	47	23.6%	16	8.1%
Interpersonal	The research team has too few members	111	55.8%	53	26.6%	35	17.6%
Methods	Researcher is not using two screeners	107	53.8%	61	30.6%	31	15.6%
Interpersonal	A student is leading the project, and the student’s faculty mentor is not helpful	98	49.2%	76	38.2%	25	12.6%
Methods	Question is defined too narrowly (i.e., search retrieves too few results to draw a conclusion)	92	46.2%	100	50.3%	7	3.5%
Methods	Researcher is not tracking reasons for exclusion	77	38.7%	58	29.1%	64	32.2%
Interpersonal	Researcher considers you only as a PDF supplier or provider of administrative tasks	76	38.2%	118	59.3%	5	2.5%
Methods	Researcher is not using two-step screening process (i.e., first reviewing title/abstract then full article)	70	35.2%	90	45.2%	39	19.6%
Methods	The researcher does not follow a data extraction plan	59	29.7%	44	22.1%	96	48.2%
Methods	Researcher does not want to evaluate study quality as part of process	56	28.1%	68	34.2%	75	37.7%
Interpersonal	The research team is dysfunctional	49	24.6%	94	47.2%	56	28.1%
Interpersonal	The research team cannot agree on question	46	23.1%	124	62.3%	29	14.6%
Interpersonal	The research team has too many members	44	22.1%	112	56.3%	43	21.6%
Interpersonal	Researcher refuses request for authorship	31	15.6%	120	60.3%	48	24.1%

Two interpersonal issues in collaborating on systematic reviews were among the common challenges: a student is leading the project with unhelpful faculty, and research team has too few members. While we presented fewer interpersonal challenges in the survey, the frequency and diversity of these issues was reflected in the free responses, where seventy-four comments referred to collaboration and interpersonal problems.

Respondents were also asked to select their top five most difficult challenges and rank them in order of difficulty. The top five challenges ranked as most difficult were: researcher does not follow systematic review methods, research question is not clear and answerable, research question is defined too broadly, inadequate inclusion and exclusion criteria, and students leading the project with unhelpful faculty.

Regression analysis was used to investigate any association between respondents reporting that they had experienced three or more of the listed methodological or interpersonal challenges sometimes or often and their reported age range, gender, years of experience as a health sciences librarian, past training in conducting systematic reviews, and the number of systematic reviews they previously worked on; however, the sample sizes within these groups were not large enough to detect statistically significant effect sizes.

Respondents had the opportunity to report other methodology and interpersonal challenges faced relating to systematic reviews that were not included in the provided lists. These results can be viewed in supplemental Appendix B. Respondents underscored many of the challenges that were included in the survey and reported several additional challenges, especially many other interpersonal challenges that were not included as part of the survey. Reported challenges were grouped into the following themes: collaboration with researcher (74 comments), adherence to systematic review methodology (31 comments), understanding of methodology (13 comments), time constraints (10 comments), and information sources (5 comments).

Challenges related to collaboration with researchers included managing their expectations with regard to time and effort associated with conducting a review as well as general communication. Respondents reported that researchers had unrealistic expectations for the time required to develop an effective search strategy as well as to complete other steps in the systematic review process. Respondents also reported that they had experienced a lack of feedback on proposed search strategies and a lack of follow-up after the search was executed on behalf of the researcher.

Another commonly reported challenge was a lack of adherence to rigorous systematic review methodologies. Respondents commonly reported issues such as not having a protocol developed prior to initiating the review, changing the protocol or research question while the review was in process, being reluctant to be comprehensive in the search strategy by not including key databases or grey literature, and limiting the search to only articles published in English.

A lack of understanding of systematic review methodology was reported among researchers as well as the librarians assisting them. In some cases, respondents felt that they did not have an adequate level of understanding of the systematic review process to allow them to fully support researchers. More often, respondents reported that researchers did not have a complete understanding of the systematic review process and that respondents spent a significant amount of time educating researchers on methodology. This seemed especially true when working with students who were conducting systematic reviews. Some respondents reported feeling frustrated that they were expected to provide training to students on database searching and systematic review methods, often with little time to do so and little help from the students’ supervisors.

Time constraints were reported as a challenge. Respondents reported difficulty balancing work on systematic reviews with other professional duties as well as an inability to keep up with the demand for support with systematic reviews at their institutions. Respondents also reported issues related to information sources used in conducting systematic reviews, including not having access to key databases and the limitations of specific databases, such as the inability to execute a complex search or export the results.

In addition to inquiring about the challenges that they experienced, respondents were also asked about the strategies that they found most helpful in overcoming challenges associated with systematic reviews. Reported strategies were grouped into the following themes: communication with researcher (82 comments), standardized procedures (15 comments), advice from colleagues (12 comments), and more experience with systematic reviews (9 comments).

The most frequent strategies reported were focused on communicating with the researcher. These strategies focused on clear and frequent communication, clarification of the role of each individual involved in the project, and in-depth in-person consultations. Many respondents also reported how useful it was to have standardized procedures. Specifically stating that using handbooks or recognized standards or requiring a form or protocol from the researcher prior to initiating the project were useful strategies. Comments related to more experience doing systematic reviews were also common. While asking more experienced colleagues for advice was one of the themes, several respondents reported that more of their own time and experience was the best strategy. An outline of potential strategies linked to particular challenges is provided in Table 3.

**Table 3:** Most frequent and most difficult challenges matched with strategies

**Type**	**Challenge**	**Suggested strategies**	**Quotes**
Methods	Research question is defined too broadly (i.e., search retrieves more results than researcher wants to screen)	In-depth consultations, educating researcher, advice from colleagues, experience	“Setting expectations more clearly at the start about how many search results will likely be returned by a broad question and learning how to talk with researchers about how to formulate a more workable, better focused question.”
Methods	Researcher does not have inclusion/exclusion criteria established at the beginning of process	In-depth consultations, guideline documents, advice from colleagues	“Clearly laying out good systematic review processes with justifications (i.e., Prisma, AMSTAR, etc.)”
Methods	Research question is not clear and answerable	In-depth consultations, advice from colleagues, experience	“Discussing the question, sometimes to death, until they figure out what they really want to find out.” “Meeting one on one with the researcher to show how a question translates into an executable search”
Methods	Researcher does not follow systematic review methodology (e.g., doing a narrative review)	Clear communication, educating researcher, guideline documents, structured service model	“I have the backing of admin to walk away if a team is using shoddy methods.” “I have found that showing them existing systematic reviews on similar topics (with clear research questions and high quality methodology) has been helpful.”
Interpersonal	A student is leading the project, and the student’s faculty mentor is not helpful.	In-depth consultations, clear communication, structured service model, educating researcher	“I found it best to have in-depth discussions with the students to tease out exactly what they are looking for. But even then, it is difficult to negotiate since they need to check with their supervisors.” “I offer to speak with the faculty mentor if necessary…if the student has time, I encourage him/her to take [a systematic review (SR)] class offered at my institution.”

## DISCUSSION

The five challenges that were ranked both as most difficult and among the top eight most frequent challenges had some common themes. Two of these challenges related to the research question, while another two related to overall methodology. The last of these five challenges was the problem of student-led projects with unhelpful faculty. These systematic review issues in particular were good topics on which to focus training and continuing education efforts.

The question refinement and general methodology challenges require an ability to think critically about the research question and negotiate with the researcher to arrive at an appropriate question or to advise the researcher that their question is not appropriate for this research methodology. Respondents suggested strategies for librarians encountering these challenges, including leading an in-depth consultation with the team, using guideline documents, sharing recognized standards, and asking more experienced colleagues. These strategies suggest librarians may benefit from additional training in how to lead an effective systematic review consultation, with guidance on research question formation and alternative research methodologies for questions that are not appropriate for a systematic review.

Student-led projects were reported as one of the most difficult and frequent challenges. This was also echoed in the free-text responses, where respondents continued to report working with students or trainees as a challenge. These responses went on to identify that communication issues were one of the main culprits for these issues, with lead faculty not responding to messages or taking too long to respond. Systematic reviews may be appealing research projects for students as they are less complex than conducting original research and are more highly regarded than narrative reviews. Systematic reviews may also be perceived as easier and faster than other types of research projects by faculty and students. Greater awareness in the research community (i.e., faculty mentors) of the characteristics of systematic reviews could help manage expectations of how much time and energy is required to complete a high-quality systematic review.

Several respondents suggested strategies to deal with student-led projects that underscored the need for a structured service model that clearly defines the terms of the provided service. Some institutions are now formalizing structured systematic review support services and requiring researchers to submit a protocol or similar agreement before they will collaborate on a systematic review [21, 22]. Elements of a good service model can include submission of a protocol up front in order to identify any methodological issues early. These protocol documents outline who has what role in the project, how many people are on the research team, what are the specific research questions and aims, who will have authorship, and what is the timeline for completion. Respondents also reported that a helpful strategy is having a culture of mentorship, where librarians can learn from their more experienced colleagues and support from library leadership to limit the service provided in cases where the research plan is not well organized. Unfortunately, there were no suggestions for how to effectively deal with the slow or lack of response to communications regarding the systematic review projects.

### Limitations

Due to the targeted nature of the screening questions in the survey, our findings might not be generalizable to all health sciences librarians, but instead focus on those with some systematic review experience. The online survey was also limited by its focused nature. The challenge options presented were not reflective of all possible challenges and were not mutually exclusive, possibly leading to some respondent bias. Similarly, the Likert-style response options (“Often,” “Sometimes,” “Rarely,” “Never”) were not defined, leaving a possibility for respondent bias. Therefore, we included open-ended response areas to help account for some of these weaknesses and allow respondents to specify their ideas of key challenges.

## CONCLUSIONS

Health sciences librarians who are engaged in supporting systematic reviews actively participate in parts of the process outside of the literature search, including educating researchers in systematic review methodology. This suggests that the role of the librarian in the systematic review process is evolving and that researchers are taking note and incorporating librarians as collaborators earlier in the process. In particular, these results suggest that the most frequent and challenging methodological issues relate to the development of the research question, and very often researchers come to librarians with inadequate research plans. Meaning that, in some cases, the librarian might have to put forth much effort to become the educator in addition to being the searcher when working on systematic reviews.

The most frequent and challenging interpersonal issues relate to communication, both with students and faculty. In many cases, these issues require the librarian to be prepared to set boundaries on collaboration. The discussed recommendations might help librarians become better equipped and have more confidence in dealing with these common issues in providing assistance with systematic reviews.

## SUPPLEMENTAL FILES

Appendix A Survey

Appendix B Free-text survey responses and coding results
