# Supplementary material for: Collaboration challenges in systematic reviews: a survey of health sciences librarians
Source: J Med Libr Assoc. 2017 Oct 1;105(4):385–93. doi: 10.5195/jmla.2017.176 (PMC5624428; doi:10.5195/jmla.2017.176)
Supplement: Appendix A [file jmla-105-385-s001.pdf]

JMLA Journal of the Medical Library Association

SC
Supplemental content to
J Med Libr Assoc. Oct;105(4): dx.doi.org/10.5195/jmla.2017.176
www.jmla.mlanet.org
© Nicholson, McCrillis, Williams 2017

Collaboration challenges in systematic reviews: a survey of health sciences librarians

Joey Nicholson, MLIS, MPH; Aileen McCrillis, MS, MPH, AHIP; Jeff Williams, MLIS, AHIP

APPENDIX A

Survey

This survey examines common issues and challenges experienced by health sciences librarians as they collaborate on systematic reviews (typically creating the search strategy and writing the methods section of the article) or provide assistance to someone else who is creating a systematic review search strategy. The survey is anonymous, so please answer fully and honestly. Thank you for your participation.

I am a librarian serving a health sciences clientele.

- o Yes
- o No

How many systematic review search strategies have you created?

- o I have only provided assistance to someone else who is creating a systematic review search strategy.
- o 1–2
- o 3–5
- o 6–8
- o More than 8
- o I have never provided assistance to someone else who is creating a systematic review search strategy or created one myself.

Considering either your experience collaborating on systematic review project(s) or assisting someone else with conducting a systematic review; how often were the following METHODS PROBLEMS encountered?

	Never	Rarely	Often	Always	Not sure
Researcher is not really doing a systematic review, e.g., doing a narrative review.	o	o	o	o	o
Researcher does not have one clear answerable question.	o	o	o	o	o
Question is defined too broadly, i.e., search retrieves more results than researcher wants to screen.	o	o	o	o	o
Question is defined too narrowly, i.e., search retrieves too few results to draw a conclusion.	o	o	o	o	o
Researcher does not have inclusion/exclusion criteria established at the beginning of process.	o	o	o	o	o
Researcher is not using two screeners.	o	o	o	o	o
Researcher is not using two-step screening process, i.e., first reviewing title/abstract then full article.	o	o	o	o	o
Researcher is not tracking reasons for exclusion.	o	o	o	o	o
Researcher does not want to evaluate study quality as part of process.	o	o	o	o	o
The researcher does not follow a data extraction plan.	o	o	o	o	o

JMLA Journal of the Medical Library Association

SC

Supplemental content to
J Med Libr Assoc. Oct;105(4): dx.doi.org/10.5195/jmla.2017.176
www.jmla.mlanet.org
© Nicholson, McCrillis, Williams 2017

What other METHODS PROBLEMS have you experienced?

Considering either your experience collaborating on systematic review projects or assisting someone else with conducting a systematic review, how often were the following INTERPERSONAL PROBLEMS encountered?

	Never	Rarely	Often	Always	Not sure
Researcher team cannot agree on question.	o	o	o	o	o
A student is leading the project, and the student's faculty mentor is not helpful.	o	o	o	o	o
Researcher refuses request for authorship.	o	o	o	o	o
The research team has too many members.	o	o	o	o	o
The research team has too few members.	o	o	o	o	o
The research team is dysfunctional.	o	o	o	o	o
Researcher considers you only as a PDF supplier or provider of administrative tasks.	o	o	o	o	o

What other INTERPERSONAL PROBLEMS have you encountered?

For the problems listed below, please drag and rank the top 5 most challenging based on your experience. 1 is most challenging, 2 is next most challenging, etc.

Items	Please select and rank the 5 most challenging problems based on your experience.
Not doing a systematic review.	
Question is not clear and answerable.	
Question is too broad.	
Question is too narrow.	
No inclusion and exclusion criteria.	
Only one screener.	
Not using two-step screening process.	
Not tracking reasons for exclusions.	
Not evaluating study quality.	
No data extraction plan.	
Team cannot agree on question.	
Student led project with unhelpful faculty mentor.	
Authorship request is refused.	
Too many team members.	
Too few team members.	
Team is dysfunctional.	
Requested to do administrative tasks.	

What has helped you the most in handling these problems over time?

Have you attended any professional development courses on systematic reviews? Please select all that apply.

- o University of Pittsburgh systematic review course
- o University of Michigan systematic review course
- o Medical Library Association webcast or webinars
- o Medical Library Association in-person continuing education course (at the annual meeting or locally)
- o Other:

JMLA Journal of the Medical Library Association

Supplemental content to
J Med Libr Assoc. Oct;105(4): dx.doi.org/10.5195/jmla.2017.176
www.jmla.mlanet.org
© Nicholson, McCrillis, Williams 2017

What is your gender?

- o Male
- o Female
- o Other:

____

- o Prefer not to say

What is your age range?

- o 20–29
- o 30–39
- o 40–49
- o 50–59
- o 60 or older
- o Prefer not to say

How many total years have you worked as a librarian serving a health sciences library clientele?

____
